# Iatrogenic Kaposi sarcoma of the terminal ileum following short-term treatment with immunomodulators for Crohn disease: A case report

**DOI:** 10.1097/MD.0000000000015714

**Published:** 2019-05-17

**Authors:** Elisa Stasi, Stefania De Santis, Elisabetta Cavalcanti, Raffaele Armentano

**Affiliations:** a Gastroenterology and Endoscopy Unit; b Laboratory of Experimental Immunopathology, National Institute of Gastroenterology “S. de Bellis,” Research Hospital Castellana Grotte, Bari; c University of Salerno, Department of Pharmacy, Fisciano (SA); d Histopathology Unit, National Institute of Gastroenterology “S. de Bellis,” Research Hospital Castellana Grotte, Bari, Italy.

**Keywords:** Crohn disease (CD), immunosuppressive therapy, Infliximab, Kaposi sarcoma

## Abstract

**Rationale:**

Kaposi sarcoma (KS) is a mesenchymal neoplasm associated with human herpes virus-8. It is often found in patients with primary or secondary immunodeficiency. An iatrogenic form of KS is detectable in patients who have received immunosuppressive therapy. To date, there are few reported cases of patients with KS treated with immunosuppressants for inflammatory bowel disease.

**Patient concerns:**

We report the case of a 45-year-old young woman with abdominal pain, episodic diarrhea and a mild weight loss. The patient was treated with immunosuppressive therapy for a parietal thickening of the terminal ileum, wrongly diagnosed as Crohn disease. After 9 months after the beginning of antitumor necrosis factor-α, the patient was admitted for obstructive symptoms. A computed tomography suspected neoplasia of ileocecal region. The patient underwent an uneventful ileocecal surgical resection.

**Diagnoses:**

The histopathology showed endometriosis of the ileal wall and an irrefutable diagnosis of KS by immunohistochemistry-positive staining for human herpes virus-8.

**Interventions and outcomes:**

The patient underwent surgical resection and is disease free at 6 years follow-up.

**Lessons:**

This case underlines the interaction of immunosuppressive therapy with the possible consequent development of visceral KS.

## 1 Introduction

Kaposi sarcoma (KS) is a vascular neoplasm with 4 variants described, each with distinct clinical and epidemiological characteristics: a classic or sporadic form, an endemic form (African), an epidemic form that is associated with human immunodeficiency virus infection and acquired immune-deficiency syndrome, and an iatrogenic form associated with immunosuppressive therapy. Although the cutaneous manifestation is the most frequent in all 4 forms, visceral localizations have also been described. The DNA sequence of a new herpes virus was identified in 1994 by Chang in tissue samples of KSs.^[1,2]^ The virus was named human herpes virus-8 (HHV-8). Subsequently, HHV-8 was detected in transplanted livers and kidneys of patients treated with immunosuppressive therapy,^[3]^ in other epidemiological variants,^[4]^ and, in some cases, also in patients with autoimmune diseases.^[5,6]^ Patients with inflammatory bowel disease (IBD), if not responding to aminosalicylates, which often represent the first line of treatment, are treated with immunomodulatory drugs. Conventional immunosuppressants such as steroids, thiopurines, and methotrexate are widely used in the treatment of IBD, as well as the more recent biologic agents, targeting specific mechanisms of the inflammatory cascade. Several cases of KS with colonic localization have been described in patients with ulcerative colitis (UC), the main risk factor being the concomitant use of immunosuppressants.^[7–10]^ The latest European guidelines for the management of opportunistic infections in patients with IBD do not mention HHV-8 in the section on herpes viruses, as data available of this infection in IBD patients are scarce.^[11]^

## 2 Case report

A 45-year-old woman presented to medical attention for abdominal pain, episodic diarrhea, and a mild weight loss (<10% body weight). Informed consent was obtained from patients included in the study. All procedures performed were in accordance with the ethical standards of our institutional research committee. She underwent a colonoscopy that showed a 4 cm stenosis of the sigmoid colon, covered with pale mucosa that could not be passed by a conventional colonscope (13.2 mm) but could be passed with an enteroscope (9.2 mm) with which the cecum could be reached; the mucosa of the other colonic tract, as well as the mucosa of the terminal ileum, were unremarkable. Random ileal and colonic biopsies were obtained, which were unremarkable as well. Histology of the stenosis showed mild inflammation, mild stromal fibrosis, and architectural disruption. Subsequently, she underwent a computed tomography scan, which showed a mild thickening of the terminal ileum and sigmoid colon. The finding of thickened terminal ileum was further confirmed by a subsequent magnetic resonance imaging enterography, suggestive for Crohn disease (CD) localized at the terminal ileum. Due to the discrepancy of the radiological findings with the initial endoscopic examination, a further colonscopy with retrograde ileoscopy was performed, showing no visible endoscopic signs of inflammation and confirming the sigmoid stenosis. The repeated ileal and colonic biopsy sampling was again not diagnostic, showing a nonspecific pattern of inflammation affecting the sigmoid colonic specimens.

The physician in charge of the patient, in view of her clinical history and the radiologic finding of intestinal thickening, reckoned that the patient was affected by CD with terminal ileum and sigmoid colon localization and proposed a therapy immunomodulators: corticosteroids 1 mg/kg for 12 weeks together with antitumor necrosis factor (TNF) infliximab 5 mg at weeks 0, 2, and 6, followed by infliximab alone. After 6 months of treatment with infliximab (5 mg/kg every 8 weeks as maintenance), an endoscopic reevaluation was scheduled, showing a resolution of the stenosis in the sigmoid colon and a normal-appearing terminal ileum. The marked improvement of the sigmoid inflammation together with the finding of fibrosis on the corresponding histology further convinced the physician in charge of the patient in favor of a diagnosis of CD, and the anti-TNF treatment with infliximab 5 mg/kg was continued. After 3 months (9 months after the beginning of anti-TNF), the patient was admitted for obstructive symptoms. A computed tomography suspected neoplasia of ileocecal region. The patient underwent an uneventful ileocecal surgical resection. The surgical specimen consisted of a portion of 8 cm of terminal ileum with the cecum and the vermiform appendix. The ileal wall showed an increased consistency and a diffuse thickening, with cystic-hemorrhagic foci in the perivisceral fat. The ileal mucosa was corrugated and showed a polypoid structure with a wide base (1.5 cm maximum diameter) originating from the submucosa and determining ulceration of the mucosal layer. The ileocecal valve, the cecum, and the appendix were normal, except for a mild lipomatous filling of the submucosa.

## 3 Histological examination

**Figure 1:**
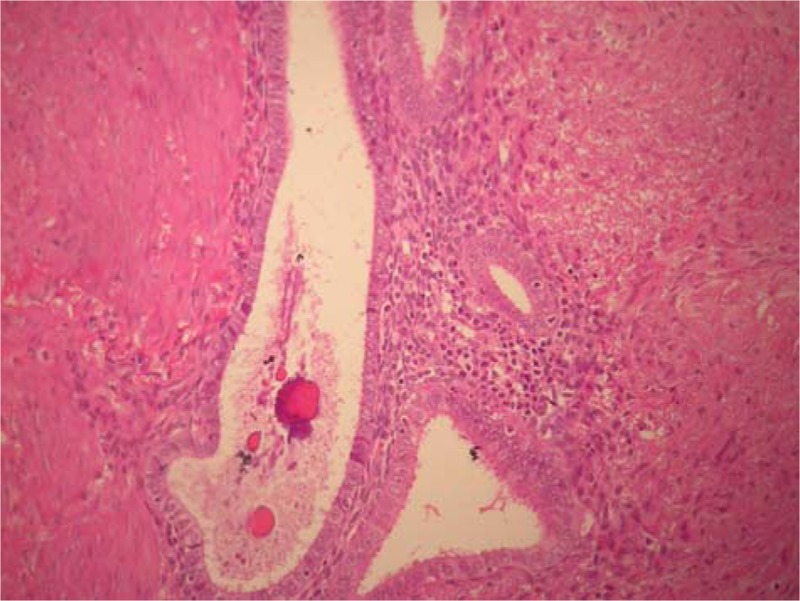
Endometriosis of the ileal wall was made (original magnification ×200).

**Figure 2:**
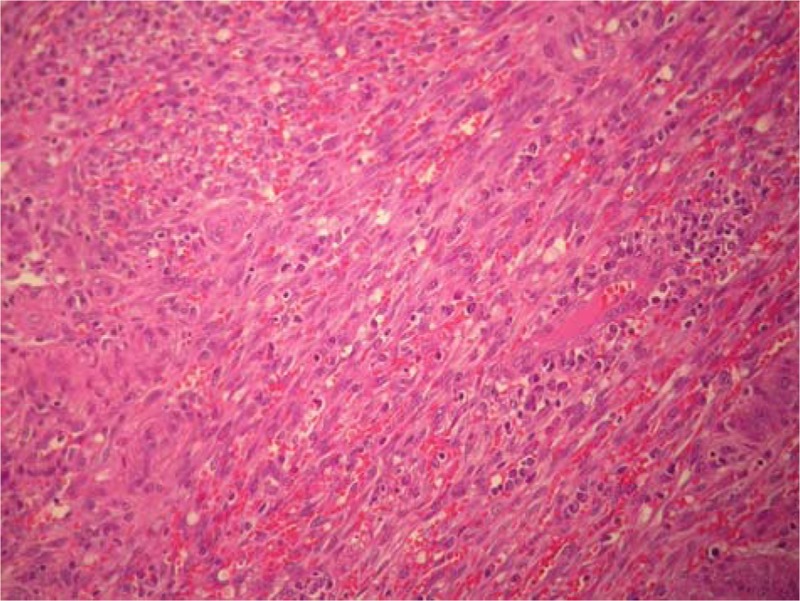
Histopathology of Kaposi sarcoma showing vascular slits surrounded by spindle cells and granulocites and midle mitoitc activity [hematoxylin and eosin (HE) staining; magnification ×200].

**Figure 3:**
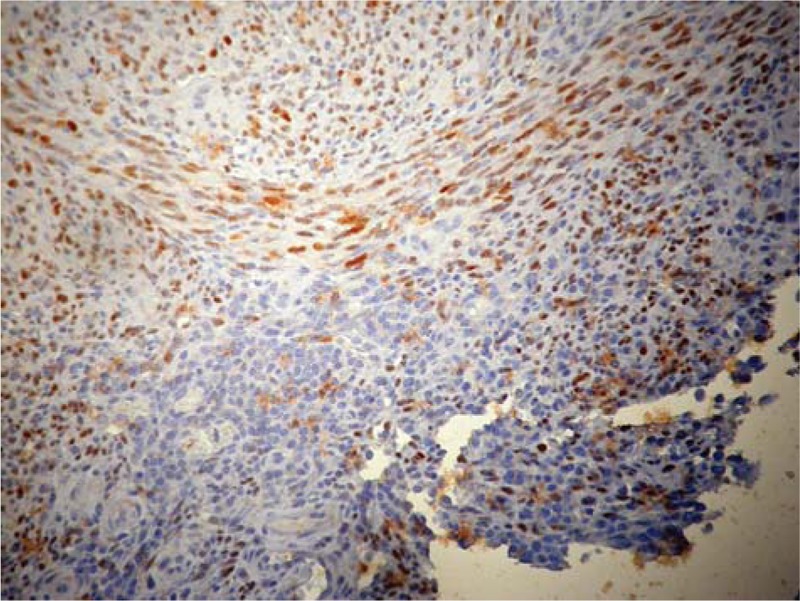
Immunohistochemistry of HHV-8 revealed diffuse nuclear staining in Kaposi sarcoma (magnification ×200). HHV-8 = human herpes virus-8.

Upon microscopic observation of the samples taken on the polypoid endoluminal lesion for intraoperative frozen section pathology, the diagnosis of endometriosis of the ileal wall was made (Fig. 1). This diagnosis was confirmed following definitive examination of the surgical specimen included in paraffin. One of the cryostate slices posed problems of differential diagnosis between a well-differentiated fusocellular sarcoma and an inflammatory pseudotumor due to endometrial heterotopy. The examination on one of the paraffin-embedded material sections unequivocally allowed the diagnosis of KS (Fig. 2). The immunohistochemical investigation for HHV-8 was positive (Fig. 3). At 6 years follow-up, the patient is disease free.

## 4 Discussion

An iatrogenic variant of KS has been described in transplant recipients treated with immunosuppressive therapy and in patients treated with immunomodulators for a broad spectrum of diseases. In transplanted patients, the incidence of KS is estimated to be between 500 and 1000 times higher than the general population.^[12]^ IBD are conditions of chronic inflammation of the gastrointestinal tract, whose overall prevalence exceeds 0.3% of the population in the Western world.^[13]^ Treatments are aimed at targeting inflammation and achieving remission, and maintaining remission in the long term. Given the prevalence and the rising incidence of the 2 main forms of IBD, UC and CD, and considering the number of patients receiving immunosuppressive therapy over the last decades, KS is a rarely reported complication to date.^[14]^ All the 4 forms of KS are associated with HHV-8 infection.^[15]^ The most common forms observed in the United States are those in association with immune-deficiency syndrome, followed by the iatrogenic forms.^[16–18]^ In the case reported in this article, the patient had received a double immunosuppressive therapy, based on corticosteroids and infliximab for a relatively short time of 12 weeks, followed by single immunosuppression with infliximab for a few months. Diagnosis of KS can be difficult in the absence of skin lesions and, although intestinal nodular lesions or polypoid masses may be visible at endoscopy, biopsies may prove negative before the tumor involves the mucosal layer.^[19]^ This case is peculiar for a variety of reasons, as the initial misinterpretation of the clinical findings led to a misdiagnosis, with a consequent incongruous therapeutic treatment. The finding of thickening of the terminal ileum was in fact a localization of endometriosis as confirmed by the pathology specimen; moreover, the sigmoid stenosis was presumably also due to a localization of endometriosis, which was in truth the real initial pathology. In fact, the diagnosis of KS was made on a patient who did not have any type of primary immunodeficiency. The interval of time during which the patient was exposed to immunosuppressive drugs was less than 1 year, whereas previous reports of KS in IBD patients regarded cases of IBD, specifically UC, treated with immunomodulators for a long time. Cetin *et al*.^[20]^ reported the case of a woman affected by UC who had been treated with azathioprine 100 mg/day for 4 years. Another case that was reported by Svrcek *et al*.^[8]^ regarded a UC patient treated with azathioprine for 19 months, preceded by long-term steroids.

The main limitation in the approach to this case is that the patient has been exposed to a double pharmacologic immunosuppression; therefore, it is not possible to differentiate the effect of steroids and the anti-TNF infliximab on the onset of iatrogenic KS. HHV-8 infection should be considered as a possible opportunistic infection not only in patients with IBD, but also in other autoimmune conditions treated with immunosuppressive drugs. The occurrence of KS in a patient with a concomitant diagnosis of IBD in the absence of an underlying immunodeficiency is fertile ground for research lines, also in vitro for the possible occurrence of rare opportunistic infections carrying oncologic implications in patients treated with immunomodulatory drugs.^[21,22]^

## 5 Conclusions

KS may arise, for reasons not currently known in detail from the biomolecular point of view, even in patients without primary immunodeficiencies or in the absence of treatments with immunomodulators protracted over long periods of time. The diagnosis, in the absence of cutaneous manifestations, can be very difficult and, especially if pictures where equivocal interpretations coexist, can be delayed. For localized forms, radical surgical treatment and withdrawal of immunomodulators alone may be considered curative.

## Author contributions

**Conceptualization:** Elisa Stasi, Raffaele Armentano.

**Data curation:** Elisa Stasi, Stefania De Santis, Elisabetta Cavalcanti.

**Formal analysis:** Stefania De Santis, Elisabetta Cavalcanti.

**Validation:** Raffaele Armentano.

**Writing – original draft:** Elisa Stasi.

**Writing – review and editing:** Raffaele Armentano, Elisabetta Cavalcanti.

Elisabetta Cavalcanti ORCID: 0000-0003-2952-0053.
